# *MicroRNA-29a* Manifests Multifaceted Features to Intensify Radiosensitivity, Escalate Apoptosis, and Revoke Cell Migration for Palliating Radioresistance-Enhanced Cervical Cancer Progression

**DOI:** 10.3390/ijms23105524

**Published:** 2022-05-15

**Authors:** Pei-Chin Chuang, Ping-Tsung Chen, Chih-Chi Wang, Wen-Hong Su, Yen-Hao Chen, Eng-Yen Huang

**Affiliations:** 1 Department of Medical Research, Kaohsiung Chang Gung Memorial Hospital, Kaohsiung 833401, Taiwan; pcjchuang@gmail.com (P.-C.C.); whsu0909@gmail.com (W.-H.S.); 2 Department of Biotechnology, Kaohsiung Medical University, Kaohsiung 807017, Taiwan; 3 Division of Hematology-Oncology, Department of Internal Medicine, Kaohsiung Chang Gung Memorial Hospital, Kaohsiung 833401, Taiwan; ptchen@cgmh.org.tw; 4 Division of General Surgery, Department of Surgery, Kaohsiung Chang Gung Memorial Hospital, Kaohsiung 833401, Taiwan; chihchiwang@cgmh.org.tw; 5 School of Medicine, College of Medicine, Chang Gung University, Taoyuan 33302, Taiwan; 6 Department of Radiation Oncology, Kaohsiung Chang Gung Memorial Hospital, Chang Gung University College of Medicine, Kaohsiung 833401, Taiwan; 7 School of Traditional Chinese Medicine, Chang Gung University, Kaohsiung 833401, Taiwan

**Keywords:** *microRNA-29a*, radioresistance, apoptosis, cell migration, cervical cancer progression

## Abstract

Radioresistance remains a major clinical challenge in cervical cancer therapy and results in tumor relapse and metastasis. Nevertheless, the detailed mechanisms are still largely enigmatic. This study was conducted to elucidate the prospective impacts of *microRNA-29a* (*miR-29a*) on the modulation of radioresistance-associated cervical cancer progression. Herein, we established two pairs of parental wild-type (WT) and radioresistant (RR) cervical cancer cells (CaSki and C33A), and we found that constant suppressed *miR-29a,* but not *miR-29b/c,* was exhibited in RR-clones that underwent a dose of 6-Gy radiation treatment. Remarkably, radioresistant clones displayed low radiosensitivity, and the reduced apoptosis rate resulted in augmented surviving fractions, measured by the clonogenic survival curve assay and the Annexin V/Propidium Iodide apoptosis assay, respectively. Overexpression of *miR-29a* effectively intensified the radiosensitivity and triggered the cell apoptosis in RR-clones. In contrast, suppressed *miR-29a* modestly abridged the radiosensitivity and abolished the cell apoptosis in WT-clones. Hence, ectopically introduced *miR-29a* into RR-clones notably attenuated the wound-healing rate and cell migration, whereas reduced *miR-29a* aggravated cell mobilities of WT-clones estimated via the in vitro wound-healing assay and time-lapse recording assay. Notably, we further established the in vivo short-term lung locomotion metastasis model in BALB/c nude mice, and we found that increased lung localization was shown after tail-vein injection of RR-CaSki cells compared to those of WT-CaSki cells. Amplified *miR-29a* significantly eliminated the radioresistance-enhanced lung locomotion. Our data provide evidence suggesting that *miR-29a* is a promising *microRNA* signature in radioresistance of cervical cancer cells and displays multifaceted innovative roles involved in anti-radioresistance, escalated apoptosis, and anti-cell migration/metastasis. Amalgamation of a nucleoid-based strategy (*miR-29a*) together with conventional radiotherapy may be an innovative and eminent strategy to intensify the radiosensitivity and further protect against the subsequent radioresistance and the potential metastasis in cervical cancer treatment.

## 1. Introduction

Cervical cancer is one of the most common gynecological malignancies in women worldwide [1]. According to the up-to-date global data released by the International Agency for Research on Cancer, there were 604,127 new diagnostic cases of cervical cancer worldwide in 2020, and 341,831 deaths appertained to cervical cancer. Radiotherapy is a well-established treatment which is used to treat more than 60% of cervical cancer cases [2]. Unfortunately, clinical evidence also indicates that 30–50% of patients with stage IB–IVA will ultimately fail following definitive radiotherapy, and the main cause of the treatment failure in patients within local cervical cancer is resistance to radiotherapy [2]. It suggests that radioresistance presents the main challenge of treatment of cervical cancer, and once it occurs, radioresistant tumors will lose the sensitivity to be salvaged by using radiotherapy again. Therefore, it has significance to reveal the mechanisms underlying radioresistance in cervical cancer. From the literature review, some progress regarding the radioresistance in cervical cancer has been proposed in the past decades. Radiation-induced p53 activation and cell cycle arrest [3], increased DNA repair of cancer cells [4], and hypoxia in the tumor microenvironment [5] have been proposed to be the major reasons for radioresistance in cervical cancer. Additionally, epidermal growth factor receptor [6], Cox-2 [7,8], and the Her-2/AKT axis [9] are also suggested in the radioresistance of cervical cancer. Nevertheless, evidence so far suggests that the mechanisms responsible for the radioresistance of cervical cancer are still largely uncertain.

The discovery of *microRNAs* (*miRNAs*) in the human genome is an important conceptual breakthrough in the post-genome sequencing era. Accumulated evidence has demonstrated that *miRNAs* have emerged as key regulators that contribute to the initiation and development of various types of cancer [10,11,12]. Radiation activates various survival and death signaling molecules, mostly involved in retraining of the cell cycle, the repair of DNA damage, and stress response-induced apoptosis, and then the rest of the viable cells will lose the radiosensitivity and form the radioresistant survivors [13,14]. Hence, the survivors of radioresistant tumors will be driven to proliferate and grow, and then the metastatic spread to secondary sites concomitant with organ failure subsequentially follows. Of late, numerous studies have demonstrated that *miRNAs* are involved in tumor growth/proliferation, differentiation, DNA repair, and apoptosis [15,16,17,18,19]. These studies support that *miRNA* maybe an attractive therapeutic strategy to deal with the radioresistant tumor progression. The *miR-29* family is comprised by three members that are encoded from two distinct genomic loci. In humans, a bicistronic transcript from chromosome 7q32.3 gives rise to *miR-29b-1* and *miR-29a*, whereas *miR-29b-2* and *miR-29c* are derived from a bicistronic transcript from chromosome 1q32.2. [19]. *miR-29b1* and *miR-29b2* sequences are identical, but they are distinguished as b1 and b2 due to the difference in locus. Recently, a growing body of evidence has also reported that *miR-29* precipitates in various cancers’ carcinogenesis and malignant transformation [20,21]. However, whether and how *miR-29* contributes to the radioresistance-enhanced cervical cancer progression is still largely enigmatic. This study was undertaken to explore whether and how the *miR-29* regulates radioresistance-enhanced cervical cancer progression, with a special focus on the investigation of the impacts of *miR-29* on the modulation of radiosensitivity after radiation treatment, radioresistance-associated apoptosis, and the subsequent cell migration/metastasis.

## 2. Results

### 2.1. Suppressed miR-29a, but Not miR-29b/c, in Radioresistant Cervical Cancer Cell Clones

**Figure 1:**
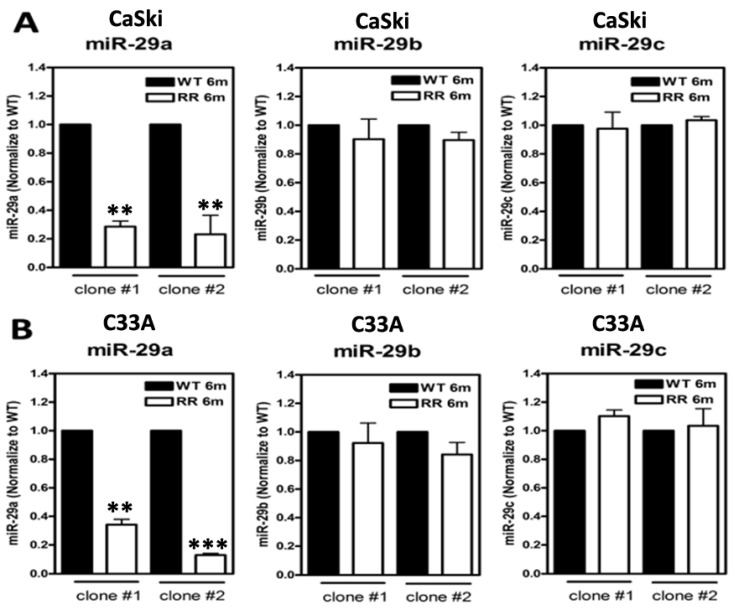
Suppressed *miR-29a* but not *miR-29b/c* in radioresistant (RR: 6 months) clones and their parental wild-type (WT) clones generated from CaSki and C33A cervical cancer cells. (**A**) Expression levels of *miR-29a/b/c* in WT and RR cervical cancer cells (CaSki) and (**B**) C33A cells. Cells were cultivated, collected, and the total *microRNA* was extracted, and expression levels of *miR-29a*, *miR-29b,* or *miR-29c* were estimated by quantitative RT-PCR analysis. The results were from two independent clones of CaSki and C33A cells, and each clone was repeated at least three times, and the results were similar. Data were shown as mean ± SD. ** *p* < 0.01, *** *p* < 0.001 for RR group vs. WT group.

We have previously already successfully established two radioresistance (RR) cervical cancer cell lines (CaSki and C33A) [22]. The detailed procedure for generating the radioresistance clones is described in Section 4.2. Two clones from CaSki and C33A cells were established, respectively. The parental cells of wild-type (WT) stable clones were generated under the same conditions without irradiation. All of the vital and stable RR colonies after 6-Gy irradiation treatments were expanded for over six months to confirm the radioresistant phenotype before mechanistic studies were undertaken. We then used these stable RR or WT cervical cancer clones to evaluate the expression levels of the three members of the *miR-29* family (*miR-29a/b/c*) (Figure 1), and we found that *miR-29a* showed the most prominent decrease in both of the individual clones of RR-CaSki cells (Figure 1A) and RR-C33A cells (Figure 1B) and then those of their parental WT cells, whereas *miR-29b/c* did not show noteworthy differences among all of the groups. This phenomenon was consistent and reproducible among the various clones of the two cell lines. Therefore, in this study, we focused on the elucidation of the role of *miR-29a* on the following studies regarding radioresistance-associated cervical tumorigenesis. We hypothesized that *miR-29a* may act as a tumor suppressor in radioresistant cervical cancer progression.

### 2.2. MicroRNA-29a Operated as an Anti-Redioresistance microRNA and Exhibited Imperative Roles in Regulation of Radiosensitivity in Radioresistant CaSki and C33A Cervical Cancer Cells

**Figure 2:**
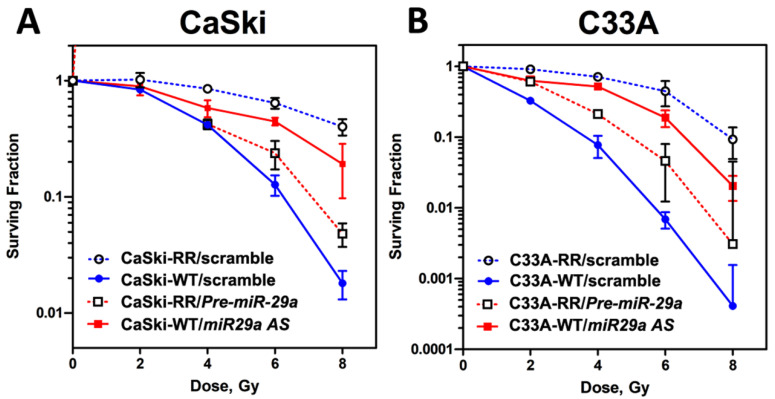
Radiation survival curve of CaSki and C33A cells performed on cells in log phase growth to evaluate the radiosensitivity. Wild-type (WT) cells were transfected with Scrambled NC (100 nM) or *miR-29a* antisense (also termed *miR-29a AS;* 100 nM)*,* whereas the radioresistant (RR) cells were transfected with Scrambled NC (100 nM) or *Pre miR-29a* precursor (also termed *Pre miR-29a;* 100 nM), respectively. Cells were plated, irradiated, and assayed for clonogenic survival at the designated dosages. All of the clonogenic assays were repeated at least three times, as described in Section 4.5. The survival curve showed the reduced radiosensitivity of the stable radioresistant clones of (**A**) CaSki and (**B**) C33A cells to irradiation compared to their parental wild-type cells. RR-cells transfected with *Pre miR-29a* reduced the surviving fraction (elevated radiosensitivity), while WT-cells transfected with *miR-29a AS* enhanced the surviving fraction (decreased radiosensitivity). Shown are mean values, and error bars show the standard deviation (SD). Solid and dotted curves represent WT and RR cells, respectively. Filled circles, WT-Scrambled NC; open circles, RR-Scrambled NC. Filled squares, WT-*miR-29a AS*; open squares, RR- *Pre miR-29a*. Each experiment was performed in triplicate and the results were similar.

Next, we sought to elucidate the impact of *miR-29a* on the modulation of the surviving populations after irradiation treatments. Firstly, we re-challenged these stable radioresistant clones with different dosages of radiation (0, 2, 4, 6, and 8 Gy) again and performed the clonogenic survival curve assay to evaluate their radiosensitivity, and the result indicated all the RR-clones of CaSki and C33A cells exhibited advanced survival rates compared to those of parental WT-clones after a dose of 6-Gy of radiation treatments (Figure 2). These data further confirmed that the surviving populations we selected as the stable RR-clones of CaSki and C33A cells were very radioresistant. Hence, we then investigated the gain or loss of function of *miR-29a* on the radiosensitivity of these WT- or RR-cell clones. Synthetic *miR-29a* precursor (Pre-miR^TM^ miRNA Precursor Molecules; *Pre-miR29a*), *miR-29a* antisense oligonucleotides (Anti-miR^TM^ miRNA inhibitor; *miR29a AS*), or scrambled negative control (Scramble NC) were transected into RR-CaSki cells or WT-CaSki cells for 72 h, respectively, as described in Section 4.4. Later, cells were subjected to various dosages of radiation treatment. After 10 to 14 days, surviving cell colonies were fixed, stained, and counted, as described in Section 4.5. As shown in Figure 2A, in WT-CaSki cells, suppression of *miR-29a* by transfection of *miR-29a* antisense oligonucleotides (*miR-29a AS* group) notably enhanced the surviving population rate (which meant decreased radiosensitivity and elevated resistance to radiation), compared to the cells transfected with scrambled negative controls (WT-Scramble NC group). In contrast, in RR-CaSki cells, overexpression of *miR-29a* by transfection of the *miR-29a* precursor (*Pre-miR29a* group) apparently reduced the surviving population rate (which meant enhanced radiosensitivity and decreased resistance to radiation). A similar phenomenon was shown in the C33A cervical cancer cells (Figure 2B). This evidence provided rationale to support that *miR-29a* was an anti-radioresistance microRNA and displayed imperative roles in the regulation of radiosensitivity.

### 2.3. MicroRNA-29a Manifested a Pro-Apoptosis Role in Radioresistant CaSki and C33A Cells

**Figure 3:**
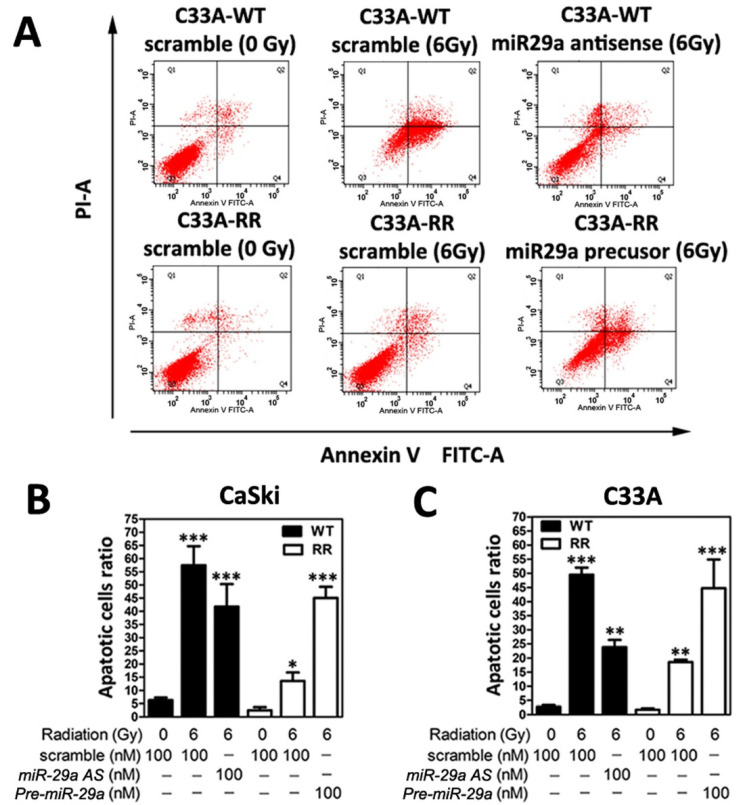
Suppressed apoptosis rate was exhibited in radioresistant CaSki and C33A cells, and overexpression of *microRNA-29a* triggered the apoptosis in radioresistant cervical cancer cells. (**A**) Representative figure of flow cytometric analysis Annexin-V/PI double staining was executed on WT- or RR-CaSki cells, which were pre-transfected with Scramble NC, *miR-29a AS,* or *Pre-miR29a*, and then were subjected to the 6-Gy radiation treatment and the apoptosis rate was estimated, as described in Section 4.6. Summary of apoptotic cells (sum of Annexin-V-positive/PI-negative and Annexin-V-positive/PI-positive cells) over 48 h after 6-Gy radiation treatment. (**B**) CaSki cells or (**C**) C33A cells. Data were obtained from three independent experiments in triplicate and shown as the mean ± SD. * *p* < 0.05, ** *p* < 0.01, *** *p* < 0.001.

Next, to confirm whether the increased surviving fraction of radioresistant CaSki and C33A cervical cancer cell clones may be due to the disruption of apoptosis, flow cytometric analysis of FITC Annexin V staining was performed, as described in Section 4.6, to evaluate the cell apoptosis rate. For quantification of the apoptotic rate, apoptotic cells were identified using the Annexin-V/FITC kit (Biouniquer, Franklin Lakes, NJ, USA), following the manufacturer’s protocol. As shown in Figure 3A, after 48 h following 6-Gy radiation treatment, the apoptotic cell ratio in the WT-CaSki scramble group was notably enhanced compared to the RR-CaSki scramble group. Furthermore, we elucidated the in vitro relevance of *miR-29a* involved in the modulation the cell apoptosis. Wild-type CaSki cells (with a relatively higher endogenous *miR-29a* level) were transiently transfected with *miR-29a AS*, and RR-CaSki cells (with a relatively lower endogenous *miR-29a* level) underwent forced introduction of *Pre-miR29a* for 72 h, respectively, and then both were subjected to the 6-Gy radiation treatment. Apparently, the results revealed that knockdown of the *miR-29a* level remarkedly reduced apoptotic cell populations in the WT-CaSki group after 48 h of 6-Gy radiation treatment (Figure 3A,B). Ectopic overexpression of *miR-29a* notably augmented the apoptotic cell populations. A consistent phenomenon was observed in the C33A cervical cancer cells (Figure 3C). Our data suggested that *miR-29a* acted as a pro-apoptosis *microRNA* to effectively trigger the apoptosis rate in radioresistant cervical cancer cells, which may lead to a beneficial effect for attenuating the radioresistant cervical cancer cell progression.

### 2.4. MicroRNA-29a Alleviated Radiosresistance-Enhanced Cell Metastatic Potential of CaSki and C33A Cells by Wound-Healing Assay and Time-Lapse Recording of Cell Movement Assay

**Figure 4:**
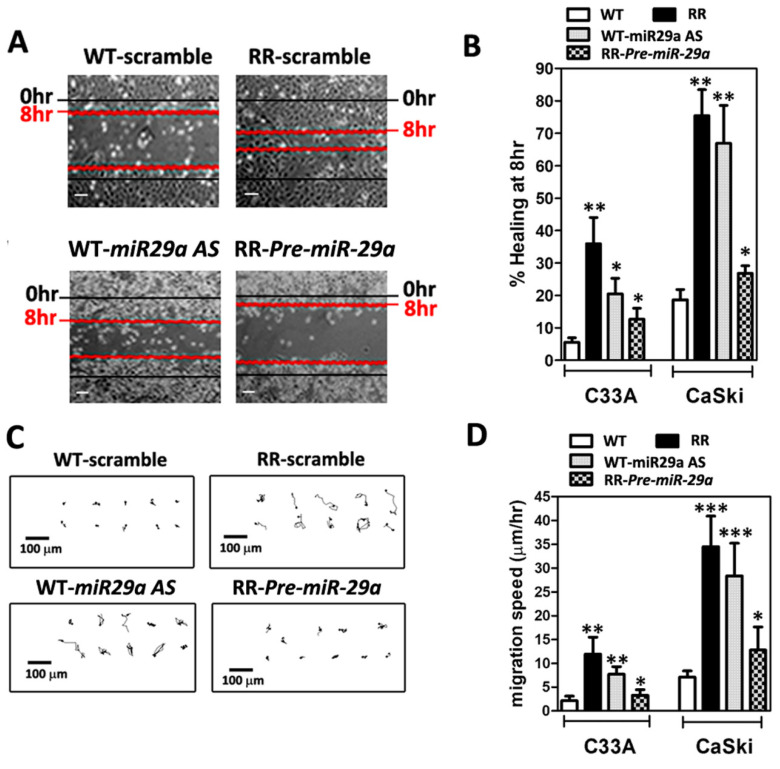
Radioresistant (RR) CaSki and C33A cells exhibited augmented migration in vitro, and overexpression of *miR-29a* effectively abrogated RR-augmented cell migration. (**A**) Representative phase-contrast micrographs depicting scratched monolayers of serum-starved WT and RR CaSki and C33A cells at 0 and 8 h. Scale bar = 100 μm. The results of each cell were repeated at least three times, and the results were similar. (**B**) Summary of wound healing 8 h after scratching. Data are shown as mean ± SD. (**C**) Representative cell migration trace from a time-lapse recording of cell migration over 8 h. The results are representative of C33A and CaSki cell clones, and each clone was repeated at least three times. Scale bar = 100 μm. (**D**) Summary of migration speed from a time-lapse recording of cell migration over 8 h. Data are the mean ± SD of three independent experiments. All radioresistant C33A and CaSki cell clones underwent stable selection for 6 months after irradiation, and >20 cells per individual sample were counted. * *p* < 0.05, ** *p* < 0.01, *** *p* < 0.001.

Radioresistance-enhanced cell metastasis is the main challenge after the failure of radiotherapy in cervical cancer [2,23]. We assessed the higher cell metastatic potential in the stable radioresistant CaSki and C33A cells, which were cultured for six months after a 6-Gy dose of irradiation treatment, and then subjected to the wound-healing assay or time-lapse recording, as described in Section 4.7 and Section 4.8. Figure 4A shows representative phase-contrast micrographs depicting scratched monolayers of serum-starved WT- and RR-CaSki cells at 0 and 8 h. We found that the radioresistant CaSki and C33A cells had persistently increased cell migration compared to their parental wild-type cells in the wound-healing assay. Furthermore, we have illustrated that in both CaSki and C33A cells, the WT-cell clones expressed relative higher *miR-29a* levels (Figure 1A,B) than those in RR-cell clones, and thus, WT-CaSki cells were transiently transfected with *miR-29a* *AS* for 72 h to reduce the *miR-29a* level. The results showed that knockdown of the *miR-29a* level significantly augmented the percent of wound-healing rate at 8 h compared with their wild-type cells (Figure 4B). In contrast, forced introduction of *Pre-miR29a* into RR-CaSki cells remarkably attenuated the wound-healing rate compared with the RR-cells group (Figure 4B). Hence, to further confirm that radioresistance was associated with augmented cell migration, time-lapse recordings of CaSki cell movement were performed hourly to trace the pathways of migrating cells (Figure 4C). Both radioresistant C33A and CaSki cells constantly migrated faster than their wild-type counterparts. Overexpression of *miR-29a* significantly diminished the increased cell migration of the radioresistant C33A and CaSki cells, whereas the reduction of *miR-29a* promoted cell migration compared with the WT-cells (Figure 4D). These data suggested that the augmented cell migration post-irradiation was steadily associated with radioresistance in C33A and CaSki cells, and *miR-29a* may act as an anti-migration-relevant *micro-RNA*.

### 2.5. Augmented Lung Locomotion after Tail-Vein Injection of RR-CaSki Cells and microRNA-29a Remarkably Abridged the Radioresistance-Enhanced Lung Locomotion in BALB/c Nude Mice

**Figure 5:**
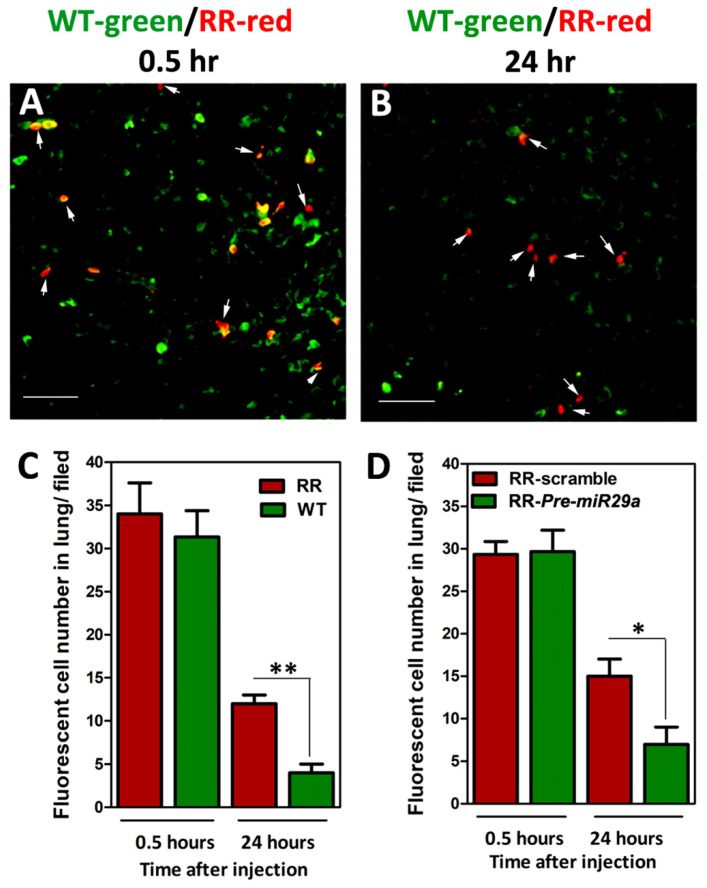
Augmented lung localization and survival rate after tail-vein injection of radioresistant (RR)-CaSki cells in BALB/c nude mice, and exogenous administration of *miR-29a* remarkedly abridged the radioresistance-enhanced lung localization. Wild-type (WT)-CaSki cells (green fluorescence) and RR-CaSki cells (red fluorescence) were mixed in a 1:1 ratio and injected into BALB/c nude mice through tail-vein injection. A representative fluorescence micrograph is shown of frozen sections of mouse lung at (**A**) 0.5 h and (**B**) 24 h post-tail-vein injection. White arrows indicate the red fluorescent RR-CaSki cells. Scale bar = 200 μm. The results were collected from six mice in each group, and the results were similar. (**C**) Summary of number of fluorescent cells in lung (red fluorescence indicating RR-CaSki cells vs. green fluorescence indicating WT-CaSki cells). (**D**) Summary of number of fluorescent cells in lung (red fluorescence indicating RR-CaSki cells transfected with Scrambled NC (100 nM) vs. green fluorescence indicating RR-CaSki cells transfected with *Pre* *miR-29a;* 100 nM). Data are the mean ± SD from six independent experiments. * *p* < 0.05, ** *p* < 0.05.

To elucidate whether the radioresistance-enhanced cervical cancer cell migrative property may lead to potent metastasis to the lung in vivo, we established the in vivo short-term lung locomotion metastasis model, as in our previous report [22]. Briefly, we injected wild-type (WT) and radioresistant (RR) CaSki cells, which had increased cell mobility, into BALB/c nude mice through the tail vein. For tracking the injected cells, CaSki cells were pre-labeled with fluorescent dyes before injection (herein, WT-CaSki cells were labeled in green fluorescence and RR-CaSki cells were labeled in red fluorescence), as described in Section 4.9. Then, the labeled cells were mixed in a 1:1 ratio and were injected into the nude mice. Mice were sacrificed at 0.5 or 24 h post-injection, and the lungs were collected and then utilized for producing frozen sections, as described in Section 4.10. As shown in Figure 5A, after 0.5 h of injection, the ratio of RR-CaSki cells (red) to WT-cells (green) retained in the lung was nearly 1:1 (after normalizing for the composition of the suspension before injection), representing that the injected cells metastasized equivalently to the lung at the early initiation time. After 24 h of injection, a notably increased number of RR-CaSki cells were retained in the lung compared to those of the WT-cell group. In a parallel experiment, RR-CaSki cells were transiently transfected with Scramble NC or *Pre-miR-29a*, respectively. Later, RR-scramble cells were labeled in red fluorescence and RR-*Pre-miR-29a* cells were labeled in green fluorescence before injection into BALB/c nude mice. Similarly, the ratio of RR-scramble (red) to RR-*Pre-miR-29a* (green) retained in the lung was nearly 1:1 after 0.5 h of injection (Figure 5D). Notably, after 24 h, the number of RR-*Pre-miR-29a* cells retained in the lung was dramatically diminished compared to the RR-scramble control group (Figure 5D). These results suggested that the radioresistance-enhanced cervical cancer cell mobility may promote the in vivo metastasis to the lung, and the forced introduced *miR-29a* effectively reduced the radioresistance-endorsed lung metastasis.

## 3. Discussion

Clinically, the identification and treatment of radioresistant cervical cancer remains an unsolved problem. The failure of radiotherapy to cure cervical cancer is frequently associated with the loss of radiosensitivity, resistance to apoptosis, uncontrolled cell growth/proliferation, and metastasis [2,23,24]. However, the detailed mechanisms remain largely elusive. This study was conducted to elucidate whether the cellular and biological impacts of *miR-29a* contribute to the augmentation of radiosensitivity, trigger apoptosis, and impede cell migration/metastasis. Herein, we have established radioresistant (RR) cervical cancer cells, RR-CaSki and RR-C33A, which were steadily expressed as suppressing the *miR-29a* level compared to their parental wild-type (WT) cells. Nevertheless, there was no significant difference in *miR-29b/c* expression between groups. We then found that the RR-clones of CaSki and C33A cells displayed higher survival rates than those of parental WT-clones after dosages (6-Gy) of irradiation treatments. Knockdown of *miR-29a* in WT-CaSki and WT-C33A cells remarkably elevated the surviving population rate (decreased the radiosensitivity) compared to the scrambled negative controls group. In contrast, forced introduction of *miR-29a* significantly abridged the surviving population rate (restored the radiosensitivity). Hence, the apoptotic cell population ratios of WT- and RR-cell clones were estimated by the Annexin V/Propidium Iodide apoptosis assay. Six-Gy radiation notably elevated the apoptotic cell ratio in both WT-CaSki and RR-CaSki cells compared to their scrambled negative control groups without irradiation treatment. Diminished *miR-29a* in WT-CaSki cells significantly decreased the apoptosis rate, whereas the overexpression of *miR-29a* in RR-CaSki cells effectively enhanced the apoptosis rate. On the other hand, we discovered that, in both CaSki and C33A, the blockage of *miR-29a* in WT-cells promoted the wound-healing rate and cell movement, while the ectopically introduced *miR-29a* into RR-cells dramatically reduced the cell mobilities. Notably, we further established the in vivo short-term lung locomotion metastasis model, and increased lung localization was shown after tail-vein injection of RR-CaSki cells compared to that in WT-CaSki cells. Overexpression of *miR-29a* significantly alleviated the radioresistance-enhanced lung locomotion in BALB/c nude mice. Taken together, our data suggested that *miR-29a* may be a specific *miRNA* signature in radioresistant cervical cancer cells and may act in a tumor-suppressive role. Additionally, our data also suggested that *miR-29a* may present various innovative roles of anti-radioresistance, pro-apoptosis, and anti-cell migration/metastasis. Ectopically expressed *miR-29a* or modulation of *miR-29a* signaling, or the combination with radiotherapy, may exhibit advantages for enhancing the radiosensitivity during radiotherapy and further preventing against subsequent radioresistance-enhanced metastasis in the future.

Radiation triggers several survival and death signaling mechanisms, including retraining of the cell cycle, DNA repair of damage, and induced apoptosis, and then the rest of the viable cell clones form the radioresistant survivors [13,14]. In clinical, impaired responsiveness of tumors to radiotherapy and uncontrolled cell growth in radioresistant tumors are the main challenges in cervical cancer treatment. Recently, several *miRNAs* have been documented to be dysregulated that influence the radiosensitivity in various cancer cells and promote cancer radioresistance. Weidhaas et al. addressed that overexpression of *let-7b* resulted in increased radiosensitivity and inhibition of *let-7g* caused radioresistance, and this activation of cell survival pathways for radioresistance was mediated by the Ras pathway in lung cancer [25]. Josson et al. have found that *miR-521* confers radiation sensitivity to LNCaP prostate cancer cells by modulating DNA repair proteins such as Cockayne syndrome protein A (CSA) and manganese superoxide dismutase (MnSOD) radiation modulation of microRNA in prostate cancer cell lines. *miR-302* enhances the sensitization of breast cancer cells to ionizing radiation [16], *miR-23b* regulates autophagy associated with radioresistance of pancreatic cancer cells [17], and *miR-205* mediates the PTEN/Akt pathway involved in both radiosensitive and radioresistant nasopharyngeal carcinoma cells [18]. Numerous studies also imply that *miRNAs* are involved in the modulation of apoptosis and cell proliferation. For example, decreased *miR-210* has been found to inhibit cell proliferation and to induce apoptosis in hypoxic human hepatoma cells [26]. *miR-221* and *miR-222* regulate gastric carcinoma cell proliferation and radioresistance by targeting PTEN [27]. *miR-214* modulates the radiotherapy response of non-small-cell lung cancer cells through regulation of cell proliferation via p38/MAPK, apoptosis, and senescence [28]. *miR-32* inhibits the proliferation of the SGC-7901 gastric cancer cell line [29], and *miR-218* impairs tumor growth and suppresses progression through downregulation of the SLIT2-ROBO1 pathway [30]. However, there is little information on whether and how *miR-29a* is involved in the modulation of radiosensitivity or whether *miR-29a* participates in the regulation of radioresistant cervical cancer progression, which need to be further explored. Thus, in this study, we first determined whether the *miR-29* family (especially *miR-29a*) may have a regulatory function in the sensitivity of radiation and radioresistance-mediated survival and apoptosis via the in vitro cellular model. A previous study has shown that the *miR-29* family is the most highly associated *miRNA* in HPV-infected cervical cancer cells [31]. HPV-related oncogene Yin Yang 1 (YY1) is a critical negative transcription factor to repress HPV16 E6/E7 promoter activity [32], and Cyclin-dependent kinase 6 (CDK6) has been identified as a regulator of HPV16 E7 protein [33]. Interesting, YY1 and CDK6 have been demonstrated as direct targets of *miR-29* by the reporter assay in rhabdomyosarcoma and lymphoma [34,35]. Clinical evidence also shown that the decreased expression of *miR-29a* in high-grade cervical cancer lesions and YY1 and CDK6 as downstream targets of *miR-29a* may contribute to unchecked cellular proliferation and resistance to apoptotic stimuli in HeLa cells [36,37]. This evidence is consistent with our finding, since we also found constant suppression of *miR-29a* in our radioresistant cervical cancer cells (Figure 1A,B). Hence, ectopic overexpression of *miR-29a* into radioresistant cells effectively blocked the survival of cell fractions after 6-Gy irradiation treatment (Figure 2A,B) and enhanced the apoptotic cell population ratio (Figure 3A,B). Our data provide imperative evidence to support that *miR-29a* may act as a vital tumor suppressor, and it effectively restricted the malignant transformation process in cervical cancer cells. Hence, we also provided reasonable clues that *miR-29a* may act as an innovative candidate *miRNA* involved in augmenting radiosensitivity and escalating the apoptotic effect to aggravate the radioresistance of cervical cancer cells.

Next, in cervical cancer, the failure of cervical cancer after radiotherapy is frequently associated with metastasis [2,23,24]. Mortality is caused mainly by metastatic spread to secondary sites, concomitant with organ failure. Therefore, we intended to investigate whether gain or loss of function of *miR-29a* affects the radioresistant-enhanced metastasis in cervical cancer. We utilized the in vitro cellular model and the in vivo BALB/c nude mice model to delineate the cellular as well as the biological contribution of *miR-29a* cells on the migration and the subsequent metastasis. As shown in Figure 4A,B, both radioresistant C33A and CaSki cells showed an increased wound-healing rate and higher cell movement compared to their wild-type counterparts, through the wound-healing assay and time-lapse recording assay, respectively. Exogenous administration of *miR-29a* dramatically reduced the increased cell mobilities of the radioresistant C33A and CaSki cells, whereas the blockage of *miR-29a* endorsed the wild-type cells’ migration (Figure 4C,D). Hence, we performed an in vivo short-term cell locomotion to the lung model with female BALB/c nude mice to test the effect of *miR-29a* on in vivo metastasis. We found a notably increased number of RR-CaSki cells retained in the lung compared to those of the WT-cells group, after 24 h of injection. Later, exogenous administration of *miR-29a* effectively interrupted the radioresistant cervical cancer cells retained in the lung compared to the RR-scramble control group (Figure 5C,D). Furthermore, we have previously demonstrated that the radioresistance-induced increase in in vitro and in vivo cell migration and metastatic properties of cervical cancer cells operates via the K-Ras/c-Raf/p38 signaling [22]. Of interest, via bioinformatics analysis, we defined that the K-Ras and c-Raf both belong to the *miR-29* target genes. Therefore, it is reasonable to suggest that the *miR-29a* may abrogate radioresistance-enhanced cell migration or lung metastasis via blockage of the K-Ras/c-Raf/p38 cascade. This evidence suggested that the radioresistance-enhanced cervical cancer cell mobility may trigger in vivo metastasis to the lung, and ectopic introduction of *miR-29a* may drive the beneficial effect to reverse the radioresistance-endorsed lung metastasis.

Furthermore, we performed the bioinformatics analysis, and numerous anti-apoptosis genes, including B cell leukemia/lymphoma 2 (BCL-2), BCL2-like 2 (BCL2L2), myeloid cell leukemia sequence 1 (MCL-1), AKT serine/threonine kinase 1 (AKT1), and so on, were predicted as miR-29a direct target genes (Supplementary Figure S3). Hence, several pro-migration genes such as CD44 molecule and MMP (matrix metalloproteinase)-7, -16, and -20 were also predicted as miR-29a direct target genes (Supplementary Figure S4). This evidence suggested that, in our radioresistant (RR) cervical cancer cells (CaSki and C33A), suppressed *miR29a* might result in a loss of the inhibition of these predicted anti-apoptosis genes (BCL-2, BCL2L2, MCL-1, and AKT1) or inhibition of pro-migration genes (CD44, MMP-7,-16, and -20), and thus RR-cell clones tended to exhibit a decreased apoptosis rate or enhanced cell-migrative properties.Taken together, of interest, the most well-documented function of *microRNA-29a* (*miR-29a*) from the literature is its role in the prevention of tissue fibrosis [38,39,40]. Our team have previously demonstrated that promotion of *miR-29a*/Wnt/DKK-1 signaling is an alternative strategy for alleviating glucocorticoid-induced bone deterioration [41]. We also further found that glucocorticoid-induced loss of miR-29a/HDAC4-mediated H3K9 acetylation signaling accelerates β-catenin deacetylation and ubiquitination, which impairs osteogenic activities of osteoblast cultures [42]. Herein, we innovatively reported that *miR-29a* may serve as a specific feature for sensitivity to radiation therapy in cervical cancer, and that targeting *miR-29* may be a novel therapeutic approach to sensitizing cervical cancer to radiation treatment and triggering the apoptosis response to diminish the radioresistant-tumor growth. Hence, we also postulated that *microRNA-29a* represents an effective treatment for interrupting the radioresistance-endorsed cell migration or potent lung metastasis. Based on our knowledge, all of these findings are novel and have never been reported before. In sum, our data provided several innovative insights and supportive evidence to suggest that a nucleoid-based *miR-29a* therapeutic strategy may intensify the radiosensitivity to radiation, reinforce the apoptosis, and exhibit the anti-migration/metastasis features to advance the radiotherapy response and prevent against subsequent metastatic potential for radioresistant cervical cancer treatment.

## 4. Materials and Methods

### 4.1. Animals

Six- to eight-week-old female BALB/c nude mice were purchased from the National Animal Center, Taiwan, and were maintained on a standard diet of chow and water ad libitum. The experimental mice were housed in an animal facility illuminated between the hours of 6:00 a.m. and 6:00 p.m. All the procedures and protocols were approved by the Institutional Animal Care and Use Committee of Chang Gung Memorial Hospital (Approval code: CGMH, 2013120503; valid period: 1 April 2013–30 September 2017).

### 4.2. Establishment of Radioresistant Cell Lines

Human cervical carcinoma cell lines CaSki and C33A were purchased from the American Type Culture Collection (ATCC, Manassas, VA, USA). The cultivated conditions and media used were those stated in the American Type Culture Collection directory. Sub-confluent cells were cultivated at 37 °C under an atmosphere of 5% CO_2_ in a T25 flask. The irradiation protocol has been described in a previous report [22]. In brief, cells were plated, cultivated, and then subjected to 6 Gy of radiation at a rate of 4 Gy per minute using a linear accelerator (Varian Medical Systems, Palo Alto, CA, USA). In a parallel experiment, one set of flasks for CaSki and C33A cells were not irradiated (defined as the wild-type group). After 24 h of cultivation, the medium in each flask was replaced with fresh medium to remove detached cells. Cells resistant to 6-Gy radiation treatment were propagated in the same culture media, and then accordingly replaced by fresh media every 3 days. All vital and stable radioresistant colonies were expanded for 180 days to confirm the radioresistant phenotype before studies were undertaken. The parental cells of wild-type stable clones for each cell type were established under identical procedures without irradiation.

### 4.3. Measurement of MicroRNA Expression by Quantitative RT-PCR

Total microRNA in cells was isolated using MicroRNA Isolation kits (BioChain Institute, Inc., Hayward, CA, USA), as described elsewhere [43]. Total microRNA was extracted and mixed with a reverse transcription (RT) mixture (Ambion, Inc., Austin, TX, USA) and subjected to reverse transcription into complementary deoxynucleic acid (cDNA). Templates of cDNA were then mixed with polymerase chain reaction (PCR) mixtures and 2× TaqMan^®^ Universal PCR Master Mix, and then PCR amplification was performed by an ABI 7900 Detection System (Applied Biosystems, Foster City, CA, USA) according to the manufacturer’s instructions. Primers of *miR-29a*, *miR29b,* and *miR29c* and endogenous control housekeeping gene *U6 small nuclear RNA* (*U6*) were purchased from Ambion, Inc. Fold change was estimated as 2^−^^ΔΔ^^Ct^, where ΔΔCt = ΔCttreatment − ΔCtsham control and ΔCt = Cttarget gene − CtU6.

### 4.4. Transfection of MicroRNA-29a Precursor and Antisense Oligonucleotide

Synthetic scrambled controls*, miR-29a precursor* (Pre-miR^TM^ miRNA Precursor Molecules), and *miR-29a* antisense oligonucleotides (Anti-miR^TM^ miRNA inhibitor) were purchased from Applied Biosystems-Ambion, Inc. (Austin, TX, USA). *Pre-miR29a* precursor molecules are designed to mimic endogenous mature *miR-29a*. *Anti-miR29a* inhibitors are designed to specifically bind to and inhibit endogenous miRNA molecules and downregulate *miR-29a* activity, as described elsewhere [41]. Cells were plated and cultivated until sub-confluence and transfected with *Pre-miR29a* precursor, *miR-29a* antisense oligonucleotide, or scrambled control by using Lipofectamine RNAiMax transfection reagent (Life Technologies, Carlsbad, CA, USA) in Opti-MEM I Reduced Serum Medium (Life Technologies) at a final concentration of 100 nM, according to the manufacturer’s instructions [44].

### 4.5. Clonogenic Survival Curve Assay after Irradiation Treatment

A clonogenic assay was performed according to the manufacturer’s instructions [22]. In brief, sub-confluent CaSki or C33A cells were cultivated in a T25 flask with the recommended medium at 37 °C under an atmosphere of 5% CO_2_. Cells (100 to 10,000 per well, according to the radiation dose) were plated in 6-well plates immediately after irradiation. After 10 to 14 days, cell colonies were fixed and stained by glutaraldehyde (6.0% *v*/*v*) and crystal violet (0.5% *w*/*v*), respectively. Colonies consisting of 50 cells were counted and defined as clonogenic survivors

### 4.6. Flow Cytometric Analysis of Annexin V/Propidium Iodide Apoptosis Assay

The Annexin V/Propidium Iodide apoptosis assay was performed according to the manufacturer’s instructions [45]. Briefly, cells were incubated with fluorescein isothiocyanate (FITC) Annexin V in a buffer containing propidium iodide (PI) and analyzed by LSR II flow cytometry (BD Biosciences, Franklin Lakes, NJ, USA). Annexin V-positive cells (sum of Annexin V-positive/PI-negative and Annexin-V-positive/PI-positive cells) were defined as apoptotic cells.

### 4.7. Wound-Healing Assay

Briefly, 1 × 10^5^/well (six-well plates) CaSki and C33A cells were cultivated as confluent monolayers and were wounded by removing a 300 to 400 μm-wide strip of cells across the well using a standard 10 μL pipette tip. Later, cells were washed, and wounded regions were healed for eight hours in serum-free medium. Wound healing was measured as the ratio of the remaining cell-free area to the area of the initial wound (estimated as a mean percentage) by ImageJ software (http://rsbweb.nih.gov/ij/index.html; accessed on 17 August 2020) according to the manufacturer’s instructions [22].

### 4.8. Time-Lapse Recording of Cell Migration

Time-lapse recording of cell movement was performed according to the manufacturer’s instructions [22]. In brief, CaSki and C33A cells were plated, and time-lapse recording was performed hourly using a microscope equipped with a camera system (Axiovert 200; Carl Zeiss MicroImaging GmbH, Welwyn Garden City, UK). Phase-contrast images were visualized using the computer software supplied with the microscope system (AxioVision; Carl Zeiss, Welwyn Garden City, UK). Images were subjected to semi-automatic tracing after every single-cell migration and movement (in µm) using AxioVision software. The migration speed was defined as the single-cell movement (in µm)/8 h.

### 4.9. Fluorescent Labeling of Cancer Cells

CaSki or C33A cells (1 × 10^7^ cells/mL) were incubated with a reduced-serum medium, Opti-MEM, containing the fluorescent CellTracker^TM^ Green CMFDA (5-chloromethylfluorescein diacetate) (Thermo Fisher Scientific, Carlsbad, CA, USA) or CellTracker^TM^ Orange CMRA Dye (10 μmol/L) for 30 min at 37 °C, according to the manufacturer’s instructions [46]. Later, cells were washed and incubated for another 30 min with dye-free PBS. After washing, the harvested cells were injected into BALB/c nude mice.

### 4.10. Locomotion of Tumor Cells to Lungs of BALB/c Nude Mice

To measure lung metastasis, as described elsewhere [46,47], wild-type and radioresistant CaSki cells were labeled with fluorescent CellTracker^TM^ Green CMFDA or CellTracker^TM^ Orange CMRA Dye (10 μmol/L), and mixed at a ratio of 1:1. A total of 2 × 10^6^ cells were injected into each mouse through the tail-vein injection. After 0.5 or 24 h of injection, lungs were harvested, and lung tissue was sectioned at 10 μm in a cryostat to observe the locomotion of fluorescent dye-stained tumor cells to the lungs. The ratio of green to red fluorescent cells in the injected suspension was counted under fluorescence microscopy (AxioVision; Carl Zeiss, Welwyn Garden City, UK).

### 4.11. Statistical Analysis

Each set of data was expressed as mean ± SD and was analyzed using one-way ANOVA using Prism software version 4.02 (GraphPad Software, San Diego, CA, USA). Dunnett’s test was applied to individually compare the results for three groups after the significance was found using the F-test. Tukey’s tests were utilized to measure whether the differences between the experimental results for paired groups were significant. The log-rank test was used to assess differences between groups of Kaplan–Meier survival curves. Student’s *t*-test was used to compare two samples.

## Data Availability

Not applicable.

## Supplementary Materials

The following supporting information can be downloaded at: https://www.mdpi.com/article/10.3390/ijms23105524/s1.
