# Supplementary material for: MicroRNA-29a Manifests Multifaceted Features to Intensify Radiosensitivity, Escalate Apoptosis, and Revoke Cell Migration for Palliating Radioresistance-Enhanced Cervical Cancer Progression
Source: Int J Mol Sci. 2022 May 15;23(10):5524. doi: 10.3390/ijms23105524 (PMC9141925; doi:10.3390/ijms23105524)
Supplement: Supplementary file 1 [file ijms-23-05524-s001.zip › ijms-1685049-supplementary.pdf]

Supplementary Figures and Legends

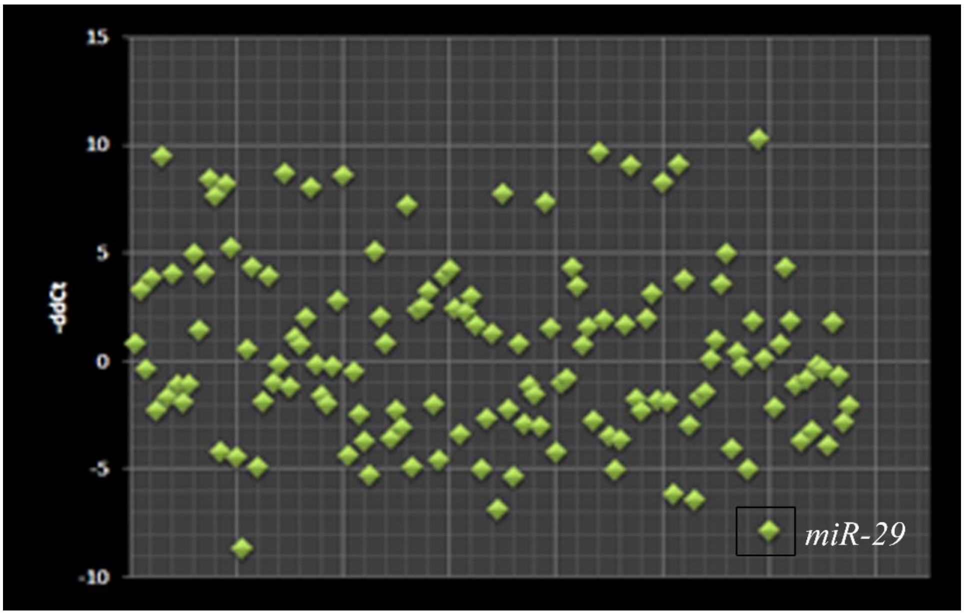

Figure S1. Representative figure of differential expression of the miRNA profiling using TaqMan Array Human MicroRNA A+B Cards Set v3.0. Total RNA was extracted from wild-type and radioresistant CaSki and C33A cells using TRIzol total RNA isolation reagent (**Thermo fisher scientific, Carlsbad, CA**). For cDNA synthesis from the miRNAs, 30 ng of total RNA was subjected to RT (reverse transcription) using a TaqMan microRNA Reverse Transcription Kit (Applied Biosystems, Foster City, CA, USA) and Megaplex Primer Pools, Human Pools Set v3.0 (Applied Biosystems, Foster City, CA, USA) following the manufacturer's protocol, allowing simultaneous reverse transcription of 754 mature human miRNAs to generate a miRNA cDNA library corresponding to each sample. After the RT step, the products were diluted, combined with TaqMan gene expression Master Mix, and then loaded into TaqMan Array Human MicroRNA A+B Cards Set v3.0 (Applied Biosystems, Foster City, CA, USA), which is a 384-well formatted array and real-time PCR-based microfluidic card with embedded TaqMan primers and probes in each well for the total 754 (A+B card) different mature human miRNAs. Real-time PCR was performed and the Ct (cycle threshold) was automatically given by SDS 2.4 software and is defined as the threshold cycle number at which the fluorescence passes the fixed threshold of 0.2. U6 snRNA embedded in the TaqMan human microRNA arrays was used as an endogenous control. The relative expression levels of miRNAs were calculated using the comparative ΔΔCt method as described in Material and Method Section 4. 3.. The fold changes in miRNAs were calculated by the equation $2^{-\Delta\Delta Ct}$. We then chose the potent target miRNAs by the following criteria (-ddCt >7 or < −7; equal to fold change >128 or <1/128), and the black square showed the *miR-29* was chosen as one of the top3 downregulated target miRNA (-ddCt < −8; equal to fold change <1/256) in radioresistant CaSki cell group while compared to the WT-group. Similar phenomena were shown in C33A cells. Each cell clone was repeated at least three times, and the results were similar. We thus performed the further

validation and study of *miR-29* and shown in Figure 1.

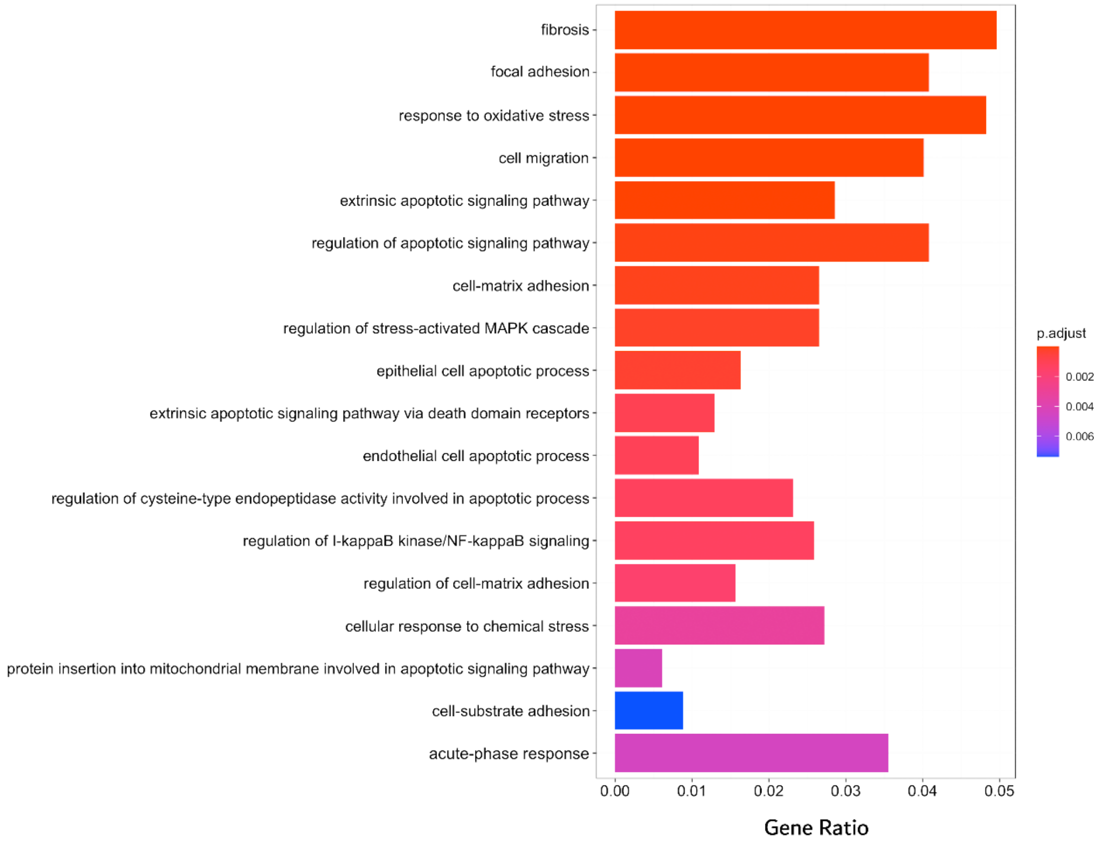

Figure S2. Bioinformatics Pathway Enrichment Analysis results of *miR-29a* target gene. Gene ontology (GO) enrichment analysis was displayed in a bar graph. Horizontal axis was GeneRatio, and the vertical axis was Gene ontology ID (GO_ID) pathways. Gene Ratio indicates that the number of differential genes/located in the GO total genes. The larger of the Gene Ratio value, indicated that the degree of GO enrichment is higher; the higher the P value was shown in redder color of the graph.

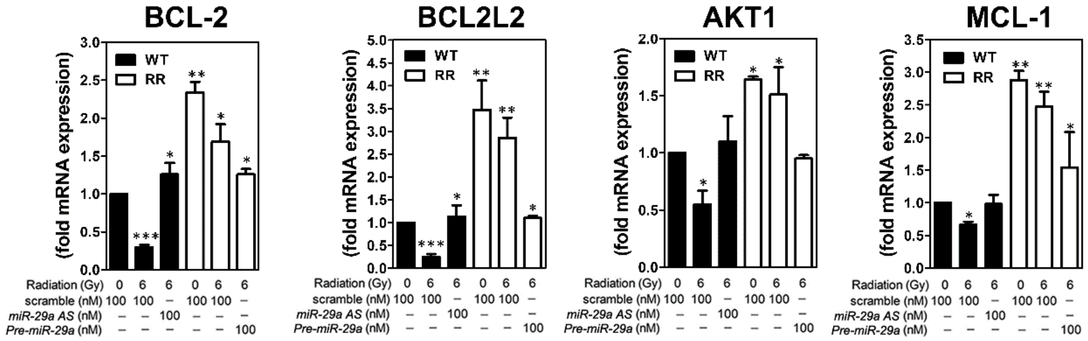

Figure S3

Figure S3. Validation of the differential expression of downstream target genes (refer to the anti-apoptosis function) regulated by *miR-29a* in CaSki cells. Wild-type (WT) or Radioresistant (RR) CaSki cells, which were pre-transfected with *scramble NC*, *miR-29a AS,* or *Pre-miR29a*, respectively, and then were subjected to the 6 Gy radiation treatment as describe in Figure 3. The RNA was extracted using an TRIzol total RNA isolation reagent (Applied Biosystems, Foster City, CA, USA). The concentration was quantified using a NanoDrop spectrophotometer. First, 1,000 ng of total RNA was subjected to reverse-transcription using a HiScript III RT SuperMix for qPCR (+gDNA wiper) (Applied Biosystems, Foster City, CA, USA) at 42°C for 2 min, 50°C for 15 min, and 85°C for 5 sec, according to the manufacturer’s instructions. The resulting cDNA was amplified using the specific 20X qPCR Primer Set for SYBR of AKT1(AKT serine/threonine kinase 1), BCL2(B cell leukemia/lymphoma 2), BCL2L2 (BCL2-like 2), MCL1(myeloid cell leukemia sequence 1) and GAPDH (GAPDH

glyceraldehyde-3-phosphate dehydrogenase) (Topgen Biotech., TW) (listed in table 1, respectively). The quantitative PCRs were performed with ChamQ Universal SYBR qPCR Master Mix (Applied Biosystems, Foster City, CA, USA) in a 10uL reaction on the 7500 FAST Real-Time PCR System (Applied Biosystems, Foster City, CA, USA) in accordance with the manufacturer's instructions. The 10ng cDNA were amplified in triplicates with appropriate non-template controls. Amplification data were normalized to GAPDH expression (shown as fold mRNA expression). Quantification of relative expression was performed using the 2-ΔΔCt relative quantification method. Quantitative PCR data showed a variability coefficient of Ct always lower than 2% of mean values. Data were obtained from three independent experiments in triplicate and are shown as the mean ± SD. * $p < 0.05$, ** $p < 0.01$, *** $p < 0.001$.

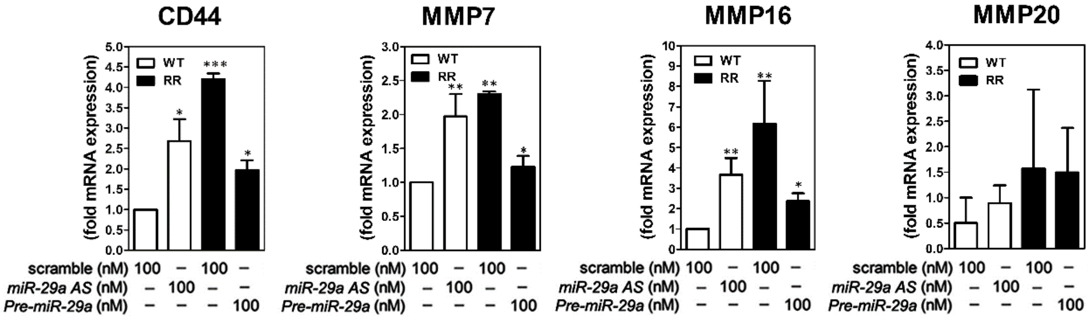

Figure S4

Figure S4. Validation of the differential expression of downstream target genes (refer to the pro-migration function) regulated by *miR-29a*. Wild-type (WT) or Radioresistant (RR) CaSki cells, which were pre-transfected with *scramble NC*, *miR-29a AS*, or *Pre-miR29a*, respectively, and then were serum-starved for 8 hours as described in Figure 4. The RNA was extracted and then subjected to reverse-transcription and the resulting cDNA was amplified using the specific 20X qPCR Primer Set for SYBR of CD44 (*CD44* Molecule), MMP (matrix metalloproteinase)- 7, -16, -20, and GAPDH (Topgen Biotech., TW) (listed in table 1, respectively). The cDNA was amplified and amplification data were normalized to GAPDH expression (shown as fold mRNA expression). ata were obtained from three independent experiments in triplicate and are shown as the mean ± SD. * $p < 0.05$, ** $p < 0.01$, *** $p < 0.001$.

Table 1. Primer list *.

No.	Gene Primer	Species	Sequence (5'-3')	RefSeq
01	AKT1-F	Human	CGAACGCACCTTCCATGTG	NM_001014431.1
	AKT1-R	Human	CCGACCGGAAGTCCATCTC	
02	BCL2-F	Human	CCTGTGGATGACTGAGTACCTGAAC	NM_000633.2
	BCL2-R	Human	CAGCCAGGAGAAATCAAACAGA	
03	BCL2L2-F	Human	ATGGAACAGGATGGGCAGAG	NM_004050.5
	BCL2L2-R	Human	CCCCTAAACTTGCCAAAACATTC	
04	MCL1-F	Human	TGGAGTTCTTCCATGTAGAGGACC	NM_182763.3
	MCL1-R	Human	AGTCAACTATTGCACTTACAGTAAGGC	
05	CD44-F	Human	CCATCTGTGCAGCAAACAACA	NM_000610.4
	CD44-R	Human	TTGGGCAGGTCTGTGACTGA	
06	MMP7-F	Human	GATTCAGCCATTTGGTGGGAGG	NM_005941.5
	MMP7-R	Human	CCCTTTCCAGACTGTGATTGGC	
07	MMP16-F	Human	TCCATCCCTGACCTCTGTGACT	NM_004771.4
	MMP16-R	Human	AGTGAACCTGCCGTCTCCAGAA	
08	MMP20-F	Human	AGTGAACCTGCCGTCTCCAGAA	NM_002423
	MMP20-R	Human	GGATCAGAGGAATGTCCCATACC	
09	GAPDH-F	Human	CTCCTCCACCTTTGACGCTG	NM_001256799.3
	GAPDH-R	Human	CTCCTTGGAGGCCATGTGG	

* All primers were designed for quantitative PCR.
